# Prevalence and Intensity of Soil-Transmitted Helminthiasis, Prevalence of Malaria and Nutritional Status of School Going Children in Honduras

**DOI:** 10.1371/journal.pntd.0003248

**Published:** 2014-10-16

**Authors:** Rosa Elena Mejia Torres, Dora Nelly Franco Garcia, Gustavo Adolfo Fontecha Sandoval, Adriana Hernandez Santana, Prabhjot Singh, Sandra Tamara Mancero Bucheli, Martha Saboya, Mirian Yolanda Paz

**Affiliations:** 1 National Parasitology Laboratory, Direction of Health Surveillance, Ministry of Health of Honduras, Tegucigalpa, Francisco Morazan, Honduras; 2 Population Risks, Ministry of Health of Honduras, Tegucigalpa, Francisco Morazan, Honduras; 3 Microbiology Research Institute, National Autonomous University of Honduras (UNAH), Tegucigalpa, Francisco Morazan, Honduras; 4 Nutrition Institute for Central America and Panama (INCAP), Tegucigalpa, Francisco Morazan, Honduras; 5 Department of Communicable Diseases and Health Analysis, Pan American Health Organization/World Health Organization (PAHO/WHO), Tegucigalpa, Francisco Morazan, Honduras; 6 Neglected, Tropical and Vector Borne Diseases Unit, Department of Communicable Diseases and Health Analysis, Pan American Health Organization/World Health Organization (PAHO/WHO), Washington D.C., United States of America; University of Kelaniya, Sri Lanka

## Abstract

**Background:**

Many small studies have been done in Honduras estimating soil-transmitted helminthiasis (STH) prevalence but a country-wide study was last done in 2005. The country has the highest burden of malaria among all Central American countries. The present study was done to estimate country-wide STH prevalence and intensity, malaria prevalence and nutritional status in school going children.

**Methods and Findings:**

A cross-sectional study was conducted following PAHO/WHO guidelines to select a sample of school going children of 3^rd^ to 5^th^ grades, representative of ecological regions in the country. A survey questionnaire was filled; anthropometric measurements, stool sample for STH and blood sample for malaria were taken. Kato-Katz method was used for STH prevalence and intensity and rapid diagnostic tests, microscopy, and polymerase chain reaction (PCR) were used for malaria parasite detection. A total of 2554 students were studied of which 43.5% had one or more STH. Trichuriasis was the most prevalent (34%) followed by ascariasis (22.3%) and hookworm (0.9%). Ecological regions II (59.7%) and VI (55.6%) in the north had the highest STH prevalence rates while IV had the lowest (10.6%). Prevalence of one or more high intensity STH was low (1.6%). *Plasmodium vivax* was detected by PCR in only 5 students (0.2%), all of which belonged to the same municipality; no *P. falciparum* infection was detected. The majority of children (83%) had normal body mass index for their respective age but a significant proportion were overweight (10.42%) and obese (4.35%).

**Conclusions:**

Biannual deworming campaigns would be necessary in ecological regions II and VI, where STH prevalence is >50%. High prevalence of obesity in school going children is a worrying trend and portends of future increase in obesity related diseases. Malaria prevalence, both symptomatic and asymptomatic, was low and provides evidence for Honduras to embark on elimination of the disease.

## Author Summary

Soil-transmitted helminthiasis (STH) are one of the most important neglected infectious diseases (NID). These not only have the potential to affect the growth and development of children, but can have irreversible consequences that carry into adulthood. However its prevalence has not been estimated since many years in Honduras. Although Honduras is the country with the highest burden of malaria in Central America, efforts to reduce the number of cases due to *Plasmodium falciparum* have lead the country towards an initiative for elimination. Using common platforms to do mapping of NID allows countries to reduce costs, build local capacities and strengthen monitoring and evaluation systems to track progress towards control and elimination goals. Honduras, one of the poorest countries in Central America, took advantage of an initiative to establish the prevalence of STH in school age children to assess the prevalence of malaria as a step in compiling evidence to support a future process of elimination of the disease. This innovative manner to put together epidemiological information for several NID might be replicated in other countries in the Americas to produce baseline and follow-up data to support decision making for processes to reach the goals established for these diseases.

## Introduction

Four nematode species are the most widely distributed and the most common in the world: *Ascaris lumbricoides*, *Trichuris trichiura*, and hookworms including *Ancylostoma duodenale* and *Necator americanus* [1]–[3]. Infestation with either one or more of these nematodes is referred to as soil-transmitted helminthiasis (STH). The Pan American Health Organization/World Health Organization (PAHO/WHO) estimates that globally almost 2 billion people are infected by schistosomiasis and STH and about 890 million children require treatment for STH [4], [5], while in the Latin American region it estimates that in 201346 million children are at risk of STH in 24 countries [6]. These estimates have been calculated based on the percentage of people without access to improved sanitation facilities, differentiated by rural and urban areas, due to the fact that STH prevalence and intensity of infection in LAC are not well mapped. The estimated disability adjusted life years (DALYs) represents a total loss of 3.8 million years due to STH globally [7]. The World Health Assembly in 2001 adopted a resolution which considers these parasites a serious public health problem [4], and in 2012 it further urged to expand interventions against these diseases [8].

As STH in children can affect their growth and development and can even lead to problems in adulthood, a multi-pronged strategy to control and prevent them involving various sections of the government and society has been advocated. Different control strategies for STH have been implemented in Latin American countries. Ecuador established a Program for Elimination of Intestinal Parasites (PEPIN) in 1994 and the Ministry of Health formed an alliance with the private organizations and volunteers involved in the deworming campaigns [9], [10]. In Honduras, deworming forms a part of the “*Escuelas Saludables*” or Healthy Schools Program which was started in 1998 and is carried out by the Ministry of Social Development. This program provides free lunch to children in public schools and carries out two deworming cycles annually in coordination with the World Food Program, among other activities, with an objective of providing an environment for adequate biological, social and emotional development of children [10]. On the other hand, data for prevalence of STH is lacking. The last country-wide prevalence study for STH was done in 2005 but was not published. The national surveillance system is not adequate for estimation of prevalence or for monitoring temporal trends of STH. Smith et al reported a prevalence of 45% for ascariasis and 38% for trichuriasis in four rural communities of the province of Francisco Morazan in Honduras in 1998 [11]. In 2005 a study to determine the intensity of STH, found that 47% of cases with severe ascariasis occurred in the province of Copan, and severe hookworm infection was found only in Copan and Colon [10]. Prevalence studies done from 2002–2006 showed an overall STH prevalence range from 12.2–97% at the province level for Honduras [12]. An unpublished study, conducted by W. Sosa, in Macuelizo valley of Santa Barbara in 2006 reported prevalence rates of 24% for ascariasis, 61% for trichuriasis and 5% for hookworm infections. More recently, a cross-sectional study among 320 school-children from Olancho reported an overall STH prevalence of 72.5% [13]. A systematic review of literature regarding the impact and prevalence of STH in Honduras during the last eight decades revealed an overall prevalence higher than 50% in more than 40% of municipalities [14].

Despite these studies and control efforts of various entities, risk factors for STH continue to be prevalent, information about STH prevalence is not available and impact of interventions not assessed. The impact of deworming component of Healthy Schools Program has never been evaluated. The Ministry of Health of Honduras carried out this study with the objective to determine the prevalence and intensity of STH in school going children, their nutritional status, and identifying local socio-demographic, cultural and environmental factors related to STH.

In Central America, Honduras is the country with the highest burden of malaria and the highest number of cases due to *Plasmodium falciparum* infection [15]. Prevention and control efforts by the national malaria program have had a big impact as evidenced by the decrease in cases, from 35,125 in 2000 to 9,628 in the year 2010. Furthermore, many municipalities have not reported a single locally acquired case of malaria over the past five years or more, laying grounds for a call towards elimination of malaria in Honduras. This study took advantage of an effort to establish baseline prevalence of STH in school children and included an assessment of the prevalence of malaria, both symptomatic and asymptomatic, among children as a step in identifying criteria for the efforts towards elimination of the disease in Honduras.

## Materials and Methods

### Ethical considerations

The study protocol was submitted to and approved by the Institutional Review Board of the Cardio-thoracic Hospital, Tegucigalpa. Written and informed consent of parents or teacher for all students assenting to participate in the study was taken 3 days prior to the survey. Either a parent or a teacher was present while the survey was being conducted and samples taken.

### Study setting and population

**Figure 1:**
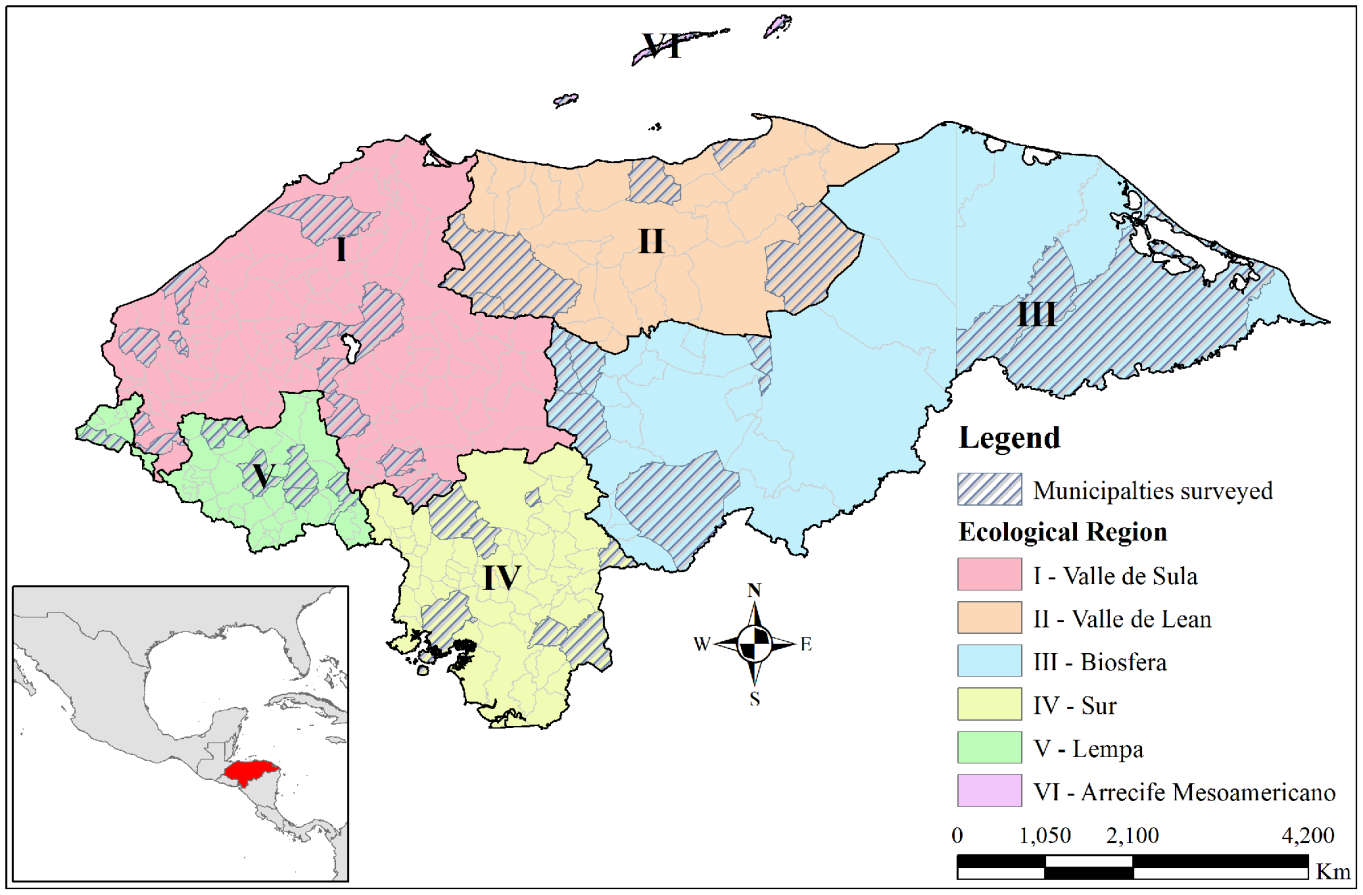
Ecological regions and municipalities selected for study, Honduras. Honduras is divided into six ecological regions. Schools from forty four different municipalities representing all the six ecological regions were selected and surveyed.

A cross-sectional study was conducted with a randomly selected sample following PAHO/WHO guidelines [16]. Fifty students of third grade and between 9 and 10 years of age per school were to be included. In schools where third grade students were less than 50 in number, fourth and fifth grade students were included; the guidelines were thus adapted to the country setting. A sample size of 2554 school going children of the third, fourth and fifth grade of 50 schools in 44 of the 298 municipalities in the country was calculated to be adequate, with a sample of at least 50 students from each school. Representation of all 18 provinces and at least 300 students from each of the six ecological regions of the country was ensured (Figure 1). Students studying in the selected schools, assenting to participate in the study and with a written and informed consent of their parents or teachers were included, while those who could not submit a stool sample on the day of the study were excluded. Children below the age of 7 and older than 14 years at the time of study were excluded.

The study was conducted under the leadership of the Sub-Secretariat of Population Risks of the Ministry of Health of Honduras and was carried out between March and December of 2011. A pilot test was done to validate the survey instruments in two schools of Francisco Morazan province, prior to their implementation. Six teams of investigators were formed and trained in the survey methodology and instruments. These teams simultaneously visited different areas of the country over a period of six weeks, each team visiting on an average 8 schools.

### Field and laboratory procedures

An individual survey questionnaire was administered to each participating student to collect demographic information (age and gender) and risk-factors (hand washing, use of slippers, and availability of drinking water and toilets at home) and history of previous deworming treatment received. Information about treatment history and water treatment method used at home was asked to the parents when present, else the child was asked. Another questionnaire for information about schools (number of students by sex, highest grade and number of teachers), their conditions (availability of water, toilets and their condition and accessibility to health center) was filled for each one visited. Principals of the school provided the answers and conditions were physically verified by the survey team. Condition of toilet was assessed to be good, regular or bad based on a subjective evaluation by the survey team.

Weight of each child was measured using electronic weighing machines after removing shoes and heavy clothes. Height was measured after removing shoe and headgears, and making the child stand alongside a wall. A container for collecting stool samples was provided to the children a day before the survey and the method of collecting the sample was explained. Stool samples were collected at home by the majority of children, and for the rest samples at school were taken; one sample per child was taken. Blood sample from finger prick was taken for evaluation of hemoglobin levels and another sample was taken on filter paper to conduct nucleic acid assays for malaria. A malaria rapid diagnostic test (Bioline, Standard Diagnostic) was done and a blood slide taken for those reporting fever on the day of the survey. At the end of the study each student received one chewable tablet of 400 mg of albendazole as deworming treatment.

The stool samples were analyzed within two hours of receipt or saved in ice where time exceeded two hours. Samples were examined in the local health center or the school using the Kato-Katz methodology for determining the prevalence and intensity of STH. Intensity of STH was analyzed as eggs per gram of stool by trained microbiologists and laboratory technicians, and was classified according to WHO guidelines (Table 1) [16]. Blood samples were analyzed for anemia using a hemoglobinometer; values below 12 mg/dl and 7 mg/dl of hemoglobin were classified as anemia and severe anemia, respectively based on guidelines followed by the Ministry of Health. Microscopic slide examination and RDTs were used for malaria diagnosis for children reporting fever, while a polymerase chain reaction (PCR) examination was carried out for all children. Detection of parasite's DNA in blood samples on filter paper was performed according to Singh et al and after a Chelex-based DNA extraction method [17], [18]. WHO Reference 2007 standard for 5 to 19 years old was used for classification of children based on body mass index (BMI) as severely thin (<−3 standard deviations), thin (<−2SD), overweight (>+1SD) and obese (>+2SD) [19], [20]. Height for age and weight for age for children less than ten years old was calculated using the WHO reference.

**Table 1:** Classification criteria for intensity of STH.

STH infection*	Severity of Infection (eggs per gram)		
	Mild	Moderate	Severe
*Ascaris lumbricoides*	1–4,999	5,000–49,999	≥50,000
*Trichuris trichiura*	1–999	1,000–9,999	≥10,000
Hookworms	1–1,999	2,000–3,999	≥4,000

* STH: Soil transmitted helminthiasis.

### Statistical analyses

Double data entry was done in Epi-info and Epi-Info, SAS and Arc-GIS were used for analysis and creating tables, graphs and maps. Anthropometric data was analyzed using Anthro-plus [21]. Univariate and multi-variate analyses of prevalence and intensity of STH was done with the socio-demographic indicators of age, sex, ecological region, risk-behaviors such as hand-washing and use of slippers, previous history of deworming treatment, drinking water and use of toilets. School level prevalence was analyzed with information on participation of the school in the Healthy Schools Program, drinking water facilities, condition of toilets and distance from the nearest health center. Prevalence of symptomatic (fever at the time of study) and asymptomatic malaria was estimated.

## Results

A little more than half of the 2554 students enrolled in the study were females and most of the students were between 8 to 11 years old (Table 2). PAHO/WHO advises to select students of third grade and between 9 and 10 year olds; however the pilot study had found many students of 7 years in 3^rd^ grade. Children up till 14 years in higher grades had to be selected to complete the sample of at least 50 per school.

**Table 2:** Children surveyed and infected according to demographic characteristics.

Category	Children surveyed	Children infected				P-value*
		Ascariasis	Trichuriasis	Hookworms	All STH	
	N (%)	%	%	%	% (95% CI)	
**Age**						0.001
7	24 (0.9%)	25.0%	37.5%	0.0%	50.0 (30.0, 70.0)	
8	393 (15.4%)	17.6%	30.3%	1.5%	36.6 (31.9, 41.4)	
9	978 (38.3%)	18.7%	32.7%	0.4%	41.1 (38.0, 44.2)	
10	635 (24.9%)	22.8%	32.1%	0.6%	42.5 (38.7, 46.4)	
11	405 (15.9%)	27.4%	39.3%	1.2%	51.1 (46.2, 56.0)	
12	96 (3.8%)	46.9%	52.1%	1.0%	64.6 (55.0, 74.2)	
13	18 (0.7%)	44.4%	33.3%	11.1%	66.7 (44.9, 88.4)	
14	5 (0.2%)	40.0%	20.0%	0.0%	40.0 (0.0, 82.9)	
**Sex**						0.011
Males	1223 (47.9%)	23.0%	35.8%	0.7%	46.1 (43.3, 48.9)	
Females	1331 (52.1%)	21.6%	32.3%	1.1%	41.1 (38.5, 43.7)	
**Ecological Region**						0.001
I	713 (27.9%)	24.0%	42.5%	0.4%	49.2 (45.6, 52.9)	
II	347 (13.6%)	28.5%	47.6%	2.3%	55.6 (50.4, 60.8)	
III	405 (15.9%)	22.5%	33.3%	0.2%	43.2 (38.4, 48.0)	
IV	360 (14.1%)	4.2%	6.9%	0.0%	10.6 (7.4, 13.7)	
V	431 (16.9%)	31.8%	17.4%	2.1%	40.8 (36.2, 45.5)	
VI	298 (11.7%)	18.8%	55.4%	0.3%	59.7 (54.2, 65.3)	
**Total**	**2554**	**569 (22.3%)**	**868 (34%)**	**22 (0.9%)**	**43.5 (41.6, 45.4)**	

*Association of demographic indicator with all children infected with STH.

STH: Soil transmitted helminthiasis; CI: Confidence Interval.

### Soil transmitted helminthiasis prevalence

**Figure 2:**
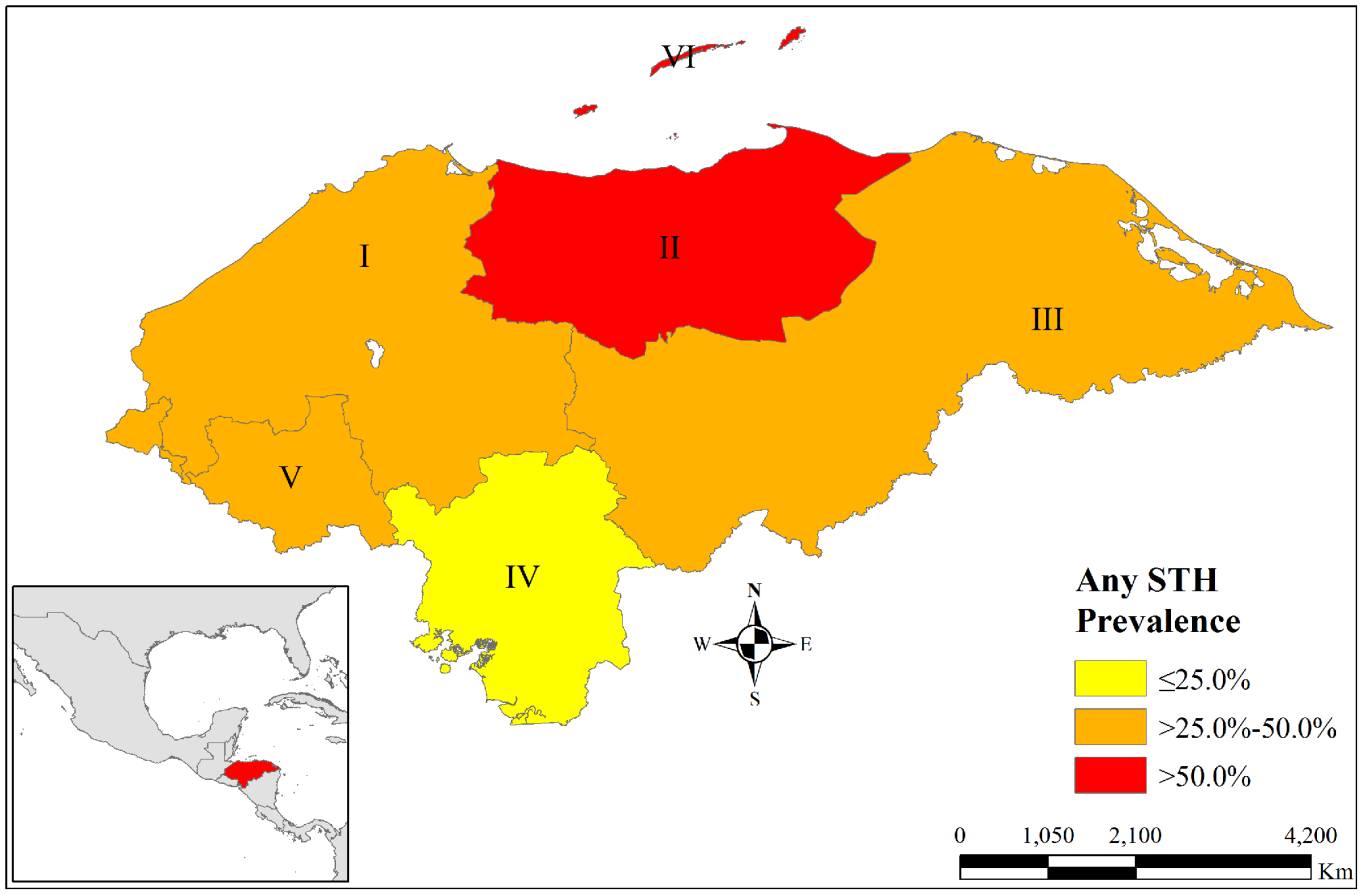
Prevalence of any STH by ecological regions, Honduras. Total prevalence of having any soil transmitted helminthiasis (STH) including ascariasis, trichuriasis or hookworm by six ecological regions of the country is shown in this map. Region II & VI had the highest prevalence while Region IV had the lowest.

Of the children studied, 43.5% had one or more STH. Trichuriasis was the most prevalent infection and affected 34% of children, ascariasis 22.3% and only 22 children had hookworm infection. *Hymenolepsis nana* (n = 22), *Strongyloides stercoralis* (n = 9) and *Enterobius vermicularis* (n = 2) were also found. Ecological region VI formed of the Bay Islands and a major tourist spot, had the highest STH prevalence (59.73%) and region II in the north of the country also had more than half the children infected (Figure 2). All other regions had more than 40% STH prevalence rates except region IV in the south of the country. Males had higher prevalence of ascariasis, trichuriasis and any STH and had 23% (1.23; 95% CI 1.05–1.44) higher odds of having STH than females. More than one STH was present in 14.6% of children (Table 3). Prevalence of one or more heavy STH was low and most children had only moderate or light STH.

**Table 3:** Children infected by severity of infection.

Type of STH infection	Severity of Infection		
	Severe	Moderate	Mild
Ascariasis	24 (0.9%)	233 (9.1%)	312 (12.2%)
Trichuriasis	20 (0.8%)	163 (6.4%)	685 (26.8%)
Hookworms	0	2 (0.1%)	20 (0.8%)
**All STH**	**40 (1.6%)**	**308 (12.1%)**	**738 (28.9%)**

STH: Soil transmitted helminthiasis.

### Anemia and nutritional status

Anemia was detected in 19.2% of children but only five children (0.2%) had severe anemia (Table 4). Using WHO reference for BMI, 2.3% children were thin or severely thin (<−2SD), but 10.4% were overweight and another 4.4% were obese. A similar pattern was seen in children up to 10 years of age, where over 12% had more than +1 SD weight-for-age and 84% were normal. Stunting (<−2SD height-for-age) was found in 11% of the children and 82% were normal. Childhood obesity was highest in region VI where almost 25% of the children were either obese or overweight followed by almost 19% in region IV. Regions II, III & VI had slightly higher proportion of children who were thin or severely thin than the national average. Two of these regions, II & VI, also had the highest prevalence of anemia. No relation was observed between thinness and prevalence of any STH (Chi-square: 3.101, p-value: 0.68).

**Table 4:** Malaria, anemia and nutritional status in children surveyed.

Ecological Region	Children surveyed	Confirmed malaria*	Anemia	Obese†	Overweight†	Thinness or severe thinness†
	N	N	N (%)	N (%)	N (%)	N (%)
I	713	0	93 (13.0%)	22 (3.1%)	68 (9.5%)	13 (1.8%)
II	347	0	148 (42.7%)	19 (5.5%)	36 (10.4%)	14 (4%)
III	405	5	47 (11.6%)	11 (2.7%)	36 (8.9%)	12 (3%)
IV	360	0	59 (16.4%)	22 (6.1%)	46 (12.8%)	6 (1.7%)
V	431	0	62 (14.4%)	10 (2.3%)	32 (7.4%)	5 (1.2%)
VI	298	0	81 (27.2%)	28 (9.4%)	48 (16.1%)	8 (2.7%)
**Total**	**2554**	**5**	**490 (19.2%)**	**112 (4.4%)**	**266 (10.4%)**	**58 (2.3%)**

*Using polymerase chain reaction.

† Obese (>+2 Standard deviations), Overweight (>+1 SD & ≤+2SD), Thin (<−2 SD and ≥−3SD) and Severe thinness (<−3 SD) according to WHO standards.

### STH risk factors

Of those responding, 95% of children washed their hands and had half the odds of having STH than those who didn't wash hands regularly (Table 5). Private tap water was the source of drinking water for more than half of the children while purified water was the second most common source. Water from wells and public taps were other frequent sources of drinking water while rain water and rain water harvesting were rare sources. Compared to those who used private tap water, children where the household used well water had significantly higher STH prevalence rates, meanwhile those using purified water had significantly lower rates. Slightly more than half of the houses of children used some form of purification method for drinking water. Chlorine tablets and boiling water were the most common methods for water purification. Children residing in houses which used boiling or other forms (except boiling and chlorine tablets) of water purification had significantly lesser odds of having STH than those where no water purification method was used, but use of chlorine tablets was not associated with any significant decline in odds of having any STH.

**Table 5:** Children surveyed and infected according to risk factors.

Category	Sub-category	Children surveyed		Children infected				Odds ratio§
		N %		Ascariasis %	Trichuriasis %	Hookworm %	All STH % (95% CI)	
Hand-washing		2518	98.6%					
	No	121	4.7%	24.0%	57.0%	1.7%	60.3 (51.6, 69.0)	*
	Yes	2394	93.7%	22.2%	32.8%	0.8%	42.6 (40.6, 44.6)	0.488†
	Sometimes	3	0.1%	0.0%	0.0%	0.0%	0.0%	
Sanitary service in house		2529	99.0%					
	No	114	4.5%	33.3%	46.5%	1.8%	57.0 (47.9, 66.1)	*
	Yes	2415	94.6%	21.4%	33.6%	0.8%	42.7 (40.8, 44.7)	0.563†
Use of toilets		2341	91.7%					
	Never	7	0.3%	42.9%	42.9%	0.0%	57.1 (20.5, 93.8)	
	Sometimes	304	11.9%	24.7%	43.4%	0.0%	50.3 (44.7, 55.9)	*
	Always	2030	79.5%	21.1%	32.4%	1.0%	42.1 (39.9, 44.2)	0.717†
Source of Drinking Water		2548	99.8%					
	Private tap water	1431	56%	24.6%	34.2%	1.1%	44.9 (42.3, 47.4)	*
	Well water	197	7.7%	44.7%	51.3%	1.0%	62.9 (56.2, 69.7)	2.088†
	Public tap water	156	6.1%	17.3%	21.8%	0.6%	35.9 (28.4, 43.4)	0.688
	Harvested rain water	22	0.9%	27.3%	36.4%	0.0%	45.5 (24.6, 66.3)	1.024
	Rain water	44	1.7%	34.1%	54.5%	0.0%	61.4 (47.0, 75.8)	1.952
	Purified	808	31.6%	13.1%	31.6%	1.0%	37.3 (33.9, 40.6)	0.730†
	Other	13	0.5%	30.8%	38.5%	0.0%	46.2 (19.1, 73.3)	1.053
Type of Treatment of Drinking water		2098	82.1%					
	None	1027	40.2%	25.1%	37.8%	0.4%	48.2 (45.1, 51.3)	*
	Chlorine tablets	411	16.1%	29.0%	38.9%	1.0%	49.4 (44.6, 54.2)	1.049
	Boiling	356	13.9%	21.1%	23.6%	1.7%	38.8 (33.7, 43.8)	0.680†
	Other	297	11.6%	16.5%	35.4%	1.7%	39.7 (34.2, 45.3)	0.708†
	Don't know	7	0.3%	14.3%	57.1%	0.0%	57.1 (20.5, 93.8)	1.433

*Reference group.

§ Association with all STH infections.

† Significant at p-value = 0.05.

Almost all (99%) children used slippers, and a majority of these always used slippers. Open slippers were more commonly used than closed slippers (Table 6). Slippers were used while going to school by almost 90% of the children, but almost or less than one-third of these used slippers at home, for playing outside or while going to church. Although history of deworming treatment was not significantly related to STH rates, children who had recently received deworming treatment (<3 months) had half the odds of having STH than those who had not received any treatment.

**Table 6:** Children surveyed and infected according to risk factors.

Category	Sub-category	Children surveyed		Children infected				Odds ratio§
		N%		Ascariasis %	Trichuriasis %	Hookworm %	All STH % (95% CI)	
Use of slippers		2546	99.7%					-
	No	24	0.9%	41.7%	29.2%	4.2%	50.0 (30.0, 70.0)	
	Yes	2522	99.1%	22%	33.9%	0.8%	43.5 (41.5, 45.4)	
Frequency of use of slippers	Always	1220	48.4%	35.1%	53.9%	1.6%	39.1 (36.4, 41.8)	-
	Sometimes	1122	44.5%	6.7%	11.8%	0.0%	46.1 (43.2, 49.0)	
Type of slippers		2364	92.6%					-
	Open	1312	55.5%	22.4%	40.6%	0.8%	47.8 (45.1, 50.5)	
	Both	286	12.1%	19.6%	31.1%	0.7%	39.5 (33.8, 45.2)	
	Closed	766	32.4%	22.6%	27.9%	0.9%	40.7 (37.3, 44.2)	
Place of using slippers		2522	98.7%					-
	Home	866	34.3%	23.1%	25.4%	0.8%	37.8 (34.5, 41.0)	
	School	2243	88.9%	22.6%	35.3%	0.8%	44.8 (42.7, 46.9)	
	Church	974	38.6%	23.6%	28.7%	0.6%	40.2 (37.2, 43.3)	
	Playing outside	773	30.7%	23.8%	26.1%	0.8%	39.2 (35.8, 42.6)	
	Others	254	10.1%	30.7%	21.7%	0.4%	40.9 (34.9, 47.0)	
History of deworming		2537	99.3%					
	No	326	12.8%	25.8%	35.9%	0.6%	47.2 (41.8, 52.7)	*
	Yes	2211	87.2%	21.7%	33.8%	0.9%	43.0 (40.9, 45.0)	0.841
Time since deworming treatment	Never	326	12.8%	25.8%	35.9%	0.6%	47.2 (47.2, 47.2)	*
	>3 months	1748	68.9%	23.9%	36.5%	1.0%	45.9 (43.6, 48.3)	0.949
	<3 months	452	17.8%	13.1%	23.5%	0.4%	31.6 (27.3, 35.9)	0.517†

*Reference group.

§ Association with all STH infections.

† Significant at p-value = 0.05.

- Association not investigated.

### School characteristics

Sixty eight per cent of the schools in the study were part of the Healthy Schools Program and 82% of all schools were within 3 kilometers of a health center (Table 7). On an average the teacher-student ratio was 1∶25 in the schools studied. All except one school had toilets, and most of these toilets were either in good or acceptable conditions. Three schools didn't have a water source and another four didn't have a potable water source. In only 56% of the schools was the water source close-by. When relating STH prevalence rates to school characteristics, it was found that children from schools included in Healthy Schools Program had 24% higher odds of having STH. Better condition of school toilets was directly related to lower odds of STH prevalence rates, as also the availability of drinking water in the school and the relative closeness of the water source to the school. Schools with better health infrastructure close-by also had lower prevalence of STH in children.

**Table 7:** School characteristics and STH prevalence rates.

School characteristics	Sub-category	Number of schools	STH Prevalence- children infected		Odds ratio§
		N	N	%	
Total Schools		50			
Number of students		385.8 (252.5)‡			
	Male	193.5 (128)‡			
	Female	192.3 (125.1)‡			
Number of teachers		15.1 (9.4)‡			
School included in Healthy School Program					
	No	16	323	39.88%	*
	Yes	34	788	45.18%	1.24†
Water source available					
	No	3	81	55.48%	*
	Yes	47	1030	42.77%	0.6†
Type of water available					
	Non-potable	7	158	47.73%	*
	Potable	40	872	41.98%	0.79†
Water source close to school					
	No	22	662	45.19%	*
	Yes	28	449	41.23%	0.85†
Type of toilet					
	None	1	34	68.00%	*
	Latrine (only)	14	129	36.24%	0.27†
	Sanitary Service (only)	28	773	43.82%	0.37†
	Both	7	175	45.57%	0.39†
Condition of toilets					
	Good	28	566	39.97%	*
	Regular	14	325	45.84%	1.27†
	Bad	7	186	49.08%	1.45†
	No toilets	1	34	68.00%	3.19†
Distance of closest health center from school (km)		1.649 (2.574)‡			
Type of Health Center					
	Hospital	4	140	72.54%	*
	Health Center with a Doctor	36	726	39.07%	0.24†
	Health Center with a Nurse	7	181	50.00%	0.38†
	Other	3	64	45.39%	0.31†

‡ Mean (Standard deviation) for continuous variables.

*Reference group.

§ Association with all soil transmitted helminthiais (STH).

† Significant at p-value = 0.05.

### Malaria prevalence

Twelve of all the students surveyed had fever at the time of the study, but all of these were negative for malaria by RDTs and by microscopy. Only 5 of the 2554 student blood samples analyzed by PCR amplification demonstrated presence of malaria parasite and all of these were *P. vivax* (Table 3). No *P. falciparum* infection was found in any sample. All these five students were asymptomatic at the time of the study and belonged to the municipality of Wampusirpi, which is known to be historically endemic for malaria, in Gracias a Dios province in the eastern part of ecological region III. No infections due to either *P. vivax* or *P. falciparum* were found using PCR as the diagnostic method in any other region of the country.

## Discussion

Prevalence rates for all STH, ascariasis and trichuriasis found in this study are similar to other previous studies but not so for hookworm infections. A study done by PAHO/WHO based on data published between 2000 and 2010 showed that 83.3% of data points found for STH prevalence for Honduras correspond to prevalence above 20% [22]. A prevalence study in Olancho province in region III in 2011 showed an overall prevalence of 72.5% for all STH, 30% for ascariasis, 67% for trichuriasis and 16% for hookworms [13].

A hospital based study of both inpatients and outpatients found hookworm prevalence rates of 0.7% and 1.5% respectively, similar to ours [23]. A study done in Atlantida (primarily Region II) and Choluteca (Region IV) in 2002 found a hookworm prevalence rate of 30–40% and 3–10% respectively in children less than 10 years of age and 40–60% and 8–10% in 10–19 year olds respectively [24]. Detection of hookworm eggs requires immediate study of stool samples which was not possible in our study owing to inadequate infrastructure in remote locations [25]. Stool samples from students had to be transported to a facility where electricity was available and in some cases could only be studied at the end of the day. This could have led to an underestimation of hookworm infection prevalence in our study.

Under the Healthy Schools Program, the Ministry of Education of Honduras in partnership with World Food Program has been deworming school age children (SAC) in schools. In 2010 it reported that 1,305,302 of an estimated 1,832,476 SAC were treated with preventive chemotherapy with a coverage of over 70% of SAC at risk which decreased to 61% in 2011 [26], [27]. However, the information regarding performance indicators such as program, geographical and national deworming coverage is not accurate. Our results show that children studying in schools included in the Healthy Schools Program had significantly higher odds of having STH. This is worrying and contrary to what is expected considering six monthly deworming campaigns in many of these schools over many years.

However, it is well documented that both host-specific and environmental factors may affect the risk of acquiring or harboring heavy-intensity helminth infections [28]. Specific occupations, household clustering, and behaviors influence the prevalence and intensity of helminth infections. STH and other helminthiases depend on environments contaminated with egg-carrying feces for transmission. Consequently, they are intimately associated with poverty, poor sanitation and lack of clean water. Due to these aforementioned factors, careful analysis of the finding of high prevalence in schools implementing the Healthy Schools Program in our study is required.

The most striking epidemiological features of human helminth infections are aggregated distributions in human communities, predisposition of individuals to heavy (or light) infection, rapid reinfection following chemotherapy, and age-intensity profiles that are typically high in children and older adults (with the exception of hookworm). The latter was also seen in our study as the odds of being infected with any STH increased by 22% (1.22; 95% CI: 1.13–1.30) for every one year increase in age of children. The rate of reinfection is specific to certain species of helminths and depends on the life expectancy of that species (short-lived helminths reinfect more rapidly), on the intensity of transmission within a given community, and on the treatment efficacy and coverage [28]. School-age children typically have the highest intensity of worm infection of any age group, and chronic infection negatively affects all aspects of children's health, nutrition, cognitive development, learning, and educational access and achievement [29]. However, the benefits of a school-based control program should also be extended to other high-risk groups (i.e. preschool children and pregnant women) and to the community at large to tackle STH prevalence [16]. A more comprehensive and coordinated approach is required in Honduras with the integration of deworming to other public health platforms to target other populations groups at risk of soil transmitted helminthic infection, as well as to integrate actions for hygiene education, water and sanitation to accelerate efforts towards STH control goals. Although the soil transmitted helminthic infections are neglected diseases that occur predominantly in rural areas, the social and environmental conditions in many unplanned slums and squatter settlements of developing countries are ideal for the persistence of *A. lumbricoides*.

If the results of our study were taken as a baseline by the Ministry of Health of Honduras in order to implement a plan to increase deworming for STH in the country, provinces and municipalities in ecological regions II and VI in the north would require targeted drug administration for STH twice a year, while regions I, III and V would require it once according to WHO guidelines [16]. Although STH prevalence in region III in the east was less than 50%, two municipalities of Gracias a Dios province had more than 90% surveyed children infected. Although the data is not representative for the province, given the low access to safe drinking water, basic sanitation and remote location, a twice-annual targeted drug administration in the province can be considered. Region IV in the south had less than 20% prevalence of any STH yet the Ministry of Health could decide to maintain the targeted drug administration at least once a year as municipalities in that region also have low rates of access to safe water and basic sanitation.

In the study done by PAHO/WHO for the period 2000–2010, 27.9% of data points found for Honduras showed heavy intensity of infection and 46.2% moderate intensity [22]. Our study found low prevalence of severe intensity infections that concurs with finding of low prevalence of anemia in the children studied. However, 12.1% of the infections were moderate. Moderate to heavy intensity infections have been documented to have significant implications for children's physical and cognitive development, as well as for severe or even fatal complications among children below 15 years of age, especially school age children [2], [30], [31]. Massive and sustained deworming has an important impact on the reduction of intensity of infection and this has a remarkably positive impact on the well-being of children [1].

Established risk factors such as hand washing, water and sewage services were significantly associated with STH prevalence rates. Most children had good hand washing habits and used toilets for their daily needs. However, water purification is done in few households and it is a continuing challenge in several parts of the country where formal public water distribution systems are still not available or are of low quality. Although almost all children replied that they used slippers and especially so while going to school, only a few used them at home or while playing outdoors. Health education interventions need to focus on promotion of use of slippers in children not only when going to school but throughout the day and in all places. Improving access to clean drinking water and good condition of toilets in schools are vital as they were directly related to lower STH prevalence. The reduction in infection prevalence will be slower if interventions rely only on deworming, as it depends on the effect of other social determinants such as access to safe water, basic sanitation, nutrition, use of footwear and housing improvement (e.g., eliminating dirt floors, adding ventilation, lighting), amongst others [1].

In 2005, Riviera found that 13.5% of school going children were overweight and 6.4% were obese in Tegucigalpa, the national capital (Region IV) [32]. In a recently concluded national demographic health survey (DHS) in 2011–12, 5.1% of children born since January 2006 were reported to be either obese or overweight [33]. The global school-based student health survey conducted in 13–15 year old students in 2012 in Honduras found 2.9%, 5.6% and 17.8% to be underweight, obese and overweight respectively [34]. Our study has found similar results in school going children. The trend of onset of obesity very early in childhood is worrying and portends of an adult population with much higher rates of obesity and associated morbidities in the near future in the country. Health and nutritional education along with physical activity programs are of utmost importance given the epidemic state of obesity in the country. On the other hand anemia in DHS was 29% in 6–59 month year olds, much higher than that in our study; however the same study also concluded that less than 1% had severe anemia similar to our results. These results reveal the precarious situation the country is in, where both under-nutrition and over-nutrition prevalence is high in children presenting significant challenges in tailoring nutritional interventions.

The very low prevalence of malaria, both symptomatic and asymptomatic, concurs with the low or no transmission of the disease in most parts of the country. All five cases of asymptomatic malaria were from the same municipality, which has historically been hyper-endemic for the disease [35]. Children sick with malaria (symptomatic) may not have attended the school on the day of the study and this could have led to an underestimation. In a malaria transmission area, asymptomatic malaria prevalence is considerable owing to immunity [36]. This study demonstrates very low prevalence rate of asymptomatic malaria carriers in children all over the country estimated using a very sensitive technique (PCR), including areas of highest malaria transmission in the country like Wampusirpi municipality (10%; 95% CI: 1.68, 18.32) in the eastern part of ecological region III. However, Honduras reports the highest number of malaria cases in Central America and the maximum number of cases are in adults. Achieving the recently declared malaria elimination goal by the council of health ministers of these countries would require surveys including residents of all ages in present or historic malaria endemic communities to find asymptomatic cases [37].

It is necessary to formulate an operational plan of action for control of STH in Honduras with the aim to integrate efforts to implement not only deworming activities but interventions to increase access to hygiene education, safe water and basic sanitation. The results of this study can be used as baseline and for monitoring the progress towards control goals of STH. The Ministry of Health of Honduras launched its national plan of action for prevention, control and elimination of nine neglected infectious diseases including STH for 2012–2017, and it is working on subnational operational plans to implement actions in priority provinces. This plan is steered by a national committee integrated by delegates of public health programs, the Ministry of Education, Non-Governmental Organizations, international organizations, donors and partners that meet monthly for monitoring the progress on the implementation of actions. Due to this national effort, deworming activities are moving forward in an integrated way since 2013, reaching pre-school age children and school age children.

As part of the monitoring of the progress towards control of STH and surveillance for malaria in the scenario of its possible elimination in Honduras, some joint activities of surveillance could be implemented in an integrated manner, for instance, through sentinel surveillance of some indicators. WHO recommends monitoring parasitological indicators for STH just before a drug administration round. Monitoring at this point will provide reliable information on reinfection occurring since the previous treatment, allowing the impact of previous treatment cycle(s) to be assessed. These data are normally collected in sentinel sites (sentinel schools) where a sample of schoolchildren should be selected in each of the ecological zones of the country [16]. Using the same platform, samples for malaria could be taken to monitor the rate of asymptomatic malaria carriers in children as part of surveillance for elimination. This is a great opportunity to improve coordination and intersectoral actions with the aims to reach STH control goals and malaria elimination.

## Supporting Information

Checklist S1 STROBE checklist.
(DOC)
